# Defining Key Features of Complex Coronary Lesions: An Evidence Based Review of Clinical Practice. Part I: Bifurcations, Left Main Disease, and Calcifications

**DOI:** 10.31083/j.rcm2306197

**Published:** 2022-05-27

**Authors:** Daniel Feldman, Frans Beerkens, Johny Nicolas, Mohan Satish, Davis Jones, James W. Johnson, George Dangas

**Affiliations:** ^1^The Zena and Michael A. Wiener Cardiovascular Institute, Icahn School of Medicine at Mount Sinai, New York, NY 10029-6574, USA; ^2^Department of Medicine, Icahn School of Medicine at Mount Sinai, New York, NY 10029-6574, USA

**Keywords:** complex percutaneous intervention, left main coronary artery disease, bifurcation lesions, calcified lesions

## Abstract

Clinicians have long recognized that certain features of coronary artery lesions increase the complexity of intervention. Complex lesions are associated with worse cardiovascular outcomes and a higher risk of subsequent ischemic events. These lesions are categorized by their angiographic features. These features include bifurcation lesions, left main coronary artery disease, calcified lesions, in-stent restenosis, chronic total occlusions and graft interventions. This two-part review aims to highlight the current evidence in the percutaneous management of these lesions. Part one of this review focuses on the best techniques to treat bifurcation lesions, indications for intervention of left main coronary artery disease and additional tools used to treat calcified lesions.

## 1. Introduction

Clinicians have long recognized that certain features of coronary artery lesions represent challenges for intervention, however defining these characteristics has remained elusive. These types of lesions were initially described by Ambrose in 1985 and classified based on their morphologic features of single vessel stenoses [1, 2]. Since then the field of angiography and coronary intervention has expanded with advancements in imaging, instrumentation and adjunctive therapeutics that have helped interventional cardiologists to perform more sophisticated procedures and intervene on more complex lesions.

**Fig. 1.**
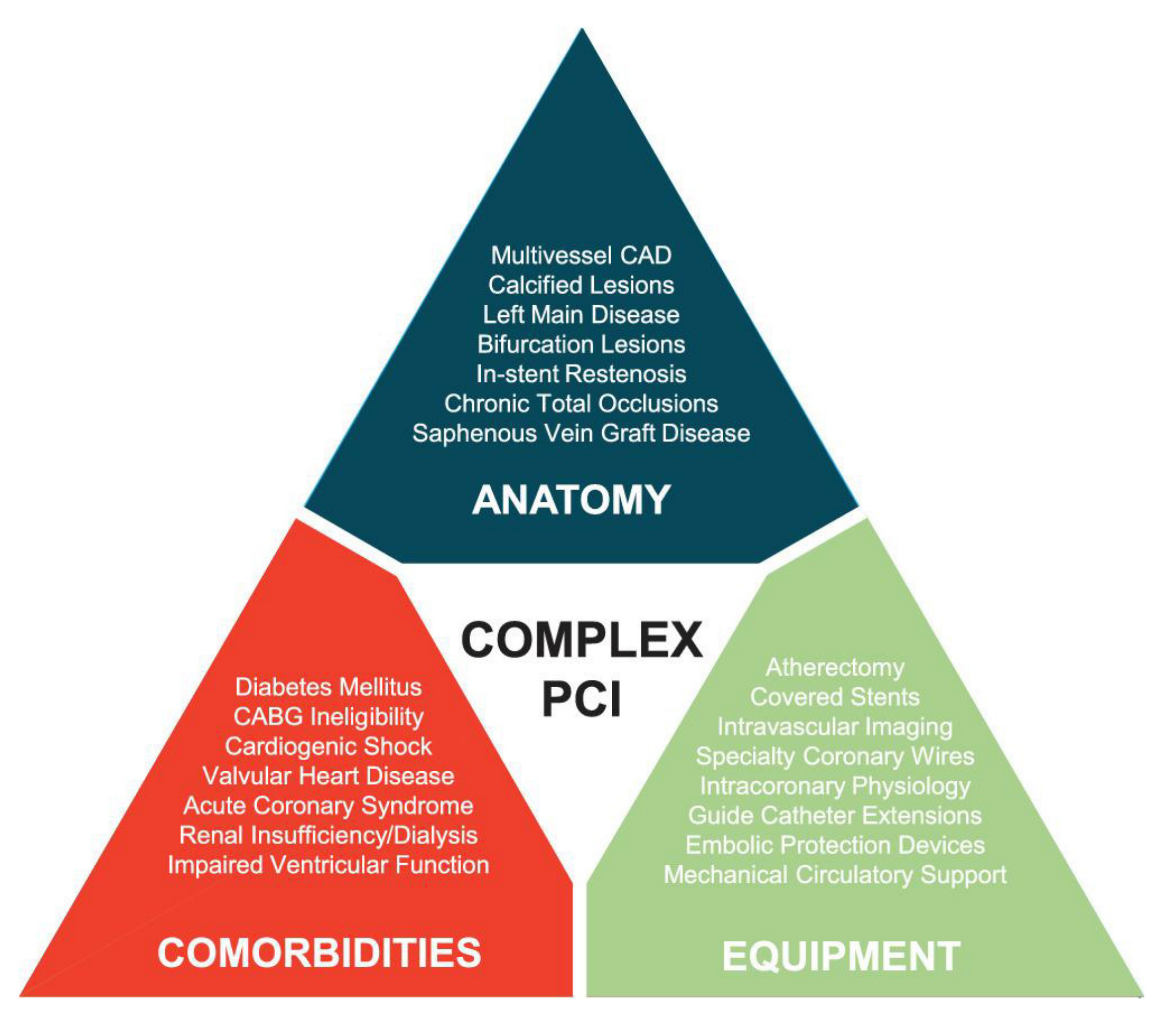
**SCAI expert consensus domains of ‘Complex PCI’, consisting of a mixture of coronary anatomy, patient co-morbidities, and interventional equipment used**. Anatomical features listed include particular lesion location and lesion morphology that add to the complexity of PCI. Comorbidities listed include underlying cardiovascular risk factors, non-cardiovascular risk factors and clinical scenarios that add to the complexity of PCI. Equipment listed include devices used to treat particular lesion morphologies, tools used to determine lesion severity and aid in lesion imaging as well as support devices used to stabilize patients, all of which add to the complexity of PCI. Reproduced with permission from Riley *et al*. [3] Copyright © 2029, John Wiley and Sons.

These advancements have resulted in differing interpretations of what is considered a “complex lesion” without a clear definition. Recently the Society for Cardiovascular Angiography and Interventions (SCAI) defined the features of ‘Complex Percutaneous Intervention (PCI)’ by three domains: anatomy, patient comorbidities, and the equipment needed (Fig. 1) [3]. This evidence-based review describes the anatomical lesions included in SCAI’s definition of a ‘Complex PCI’. The goal of this review is to highlight the characteristics of each of these anatomical lesions which are classified as ‘complex’ and to discuss the most relevant controversies regarding the classification of each specific lesion. In addition, adjunctive tools and antiplatelet strategies to help operators navigate these ‘complex’ lesions are reviewed. Recommendations for specific intervention techniques and treatment algorithms to achieve optimal results are also provided. In the first part of this two-part comprehensive review; bifurcation lesions, left main coronary artery disease and calcified lesions are examined. The focus of part two will be on chronic total occlusions, graft interventions, in-stent restenosis and antiplatelet strategies.

## 2. Alternative Definitions of Complexity 

Currently, there is no single definition of complex coronary lesions that is universally accepted. Alternative definitions to the one put forward by SCAI that are used in clinical research often include procedural characteristics. One set of criteria includes 3+ vessels treated, >/ = 3 stents implanted, >/ = 3 lesions treated, bifurcations with 2 stents implanted, total stent length >60 mm, or chronic total occlusion. These criteria were shown to be associated with higher ischemic risk in a graded fashion and these criteria have been adopted by others and have since been validated [4, 5]. While these criteria are often employed, other similar definitions are also used in clinical research; resulting in a lack of universal agreement [6].

An alternative lesion classification is from the American College of Cardiology/American Heart Association (ACC/AHA), which uses angiographic features to predict the rate of procedural success. Lesions classified as Type B2 and C are associated with lower success and higher risk and are included as complex lesions [7].

The SYNTAX score and the newly developed SYNTAX II score are other systems that categorize lesion complexity [8, 9]. They are both based on the well-known left main coronary artery and multivessel coronary artery disease (CAD) trial. The benefits of the newer scoring system are that it combines angiographic features with clinical characteristics and can help guide decision making for left main coronary artery disease (LMCAD). However, outside of this clinical indication, this scoring system is less well validated.

Lastly, there is a growing trend to define complex PCI by the clinical scenario and patient co-morbidities in addition to procedural characteristics. SCAI has incorporated these domains into their definition. An alternative term used that incorporates both clinical features and procedural characteristics to define high-risk scenarios is ‘complex high-risk indicated percutaneous coronary intervention’ (CHIP-PCI). Recently a scoring system to define CHIP-PCI has been derived to categorize the level of procedural risk that includes both clinical and procedural characteristics and has been shown to correlate with adverse events [10].

## 3. Bifurcation Lesions

Bifurcation lesions are commonly encountered and represent 15–20% of all PCIs [11]. These lesions are associated with a lower procedural success rate and an increased rate of long term complications including in-stent thrombosis and restenosis [11, 12]. The ACC/AHA task force classifies these lesions as “coronary stenosis involving a bifurcation or branch point of a vessel into at least two branches, each of which is ≥1.5 mm in diameter” while the European Bifurcation Club (EBC) defines these lesions as “a coronary artery narrowing occurring adjacent to, and/or involving, the origin of a significant side branch” [13, 14].

These lesions can be technically challenging due to the clinical setting and anatomy of the branches as well as the morphology of the disease within each branch. Specific clinical and anatomical circumstances include the vessel size and length, the amount of myocardium supplied, and whether there is collateralization of the bifurcation lesion. Specific plaque considerations include the presence of thrombosis or calcium [15]. Additional challenges include the bifurcation angle, carinal shift during the procedure, differences in vessel diameter, and technical challenges when attempting to deploy two stents if necessary.

Due to the complexity of the intervention, classification systems have been established to guide the interventionalist as to how to approach this heterogenous group. The most commonly accepted classification system is the Medina classification, which uses a binary system to categorize stenotic narrowing of >/ = 50% in each of the three arterial segments that make up the bifurcation in the order of first the proximal main vessel, then the distal main vessel, and finally the side branch [16]. While the Medina classification has benefits in its simplicity, it lacks information on the length and angulation of the lesion which can affect the technique that is used.

### 3.1 Stent Techniques 

Treatment for bifurcation lesions include a provisional one stent strategy and a dedicated up-front two-stent approach. In the provisional one stent strategy, both the main vessel and side branch are wired and only the main vessel is intervened upon with a DES. Following stent insertion, the proximal optimization technique (POT) is used to ensure main vessel stent apposition in the relatively increased proximal vessel diameter and to facilitate a larger strut opening in the side branch to allow for guidewire exchange while minimizing carinal shift [15]. The provisional stent technique is usually successful without the need for a rescue stent in the side branch.

**Fig. 2.**
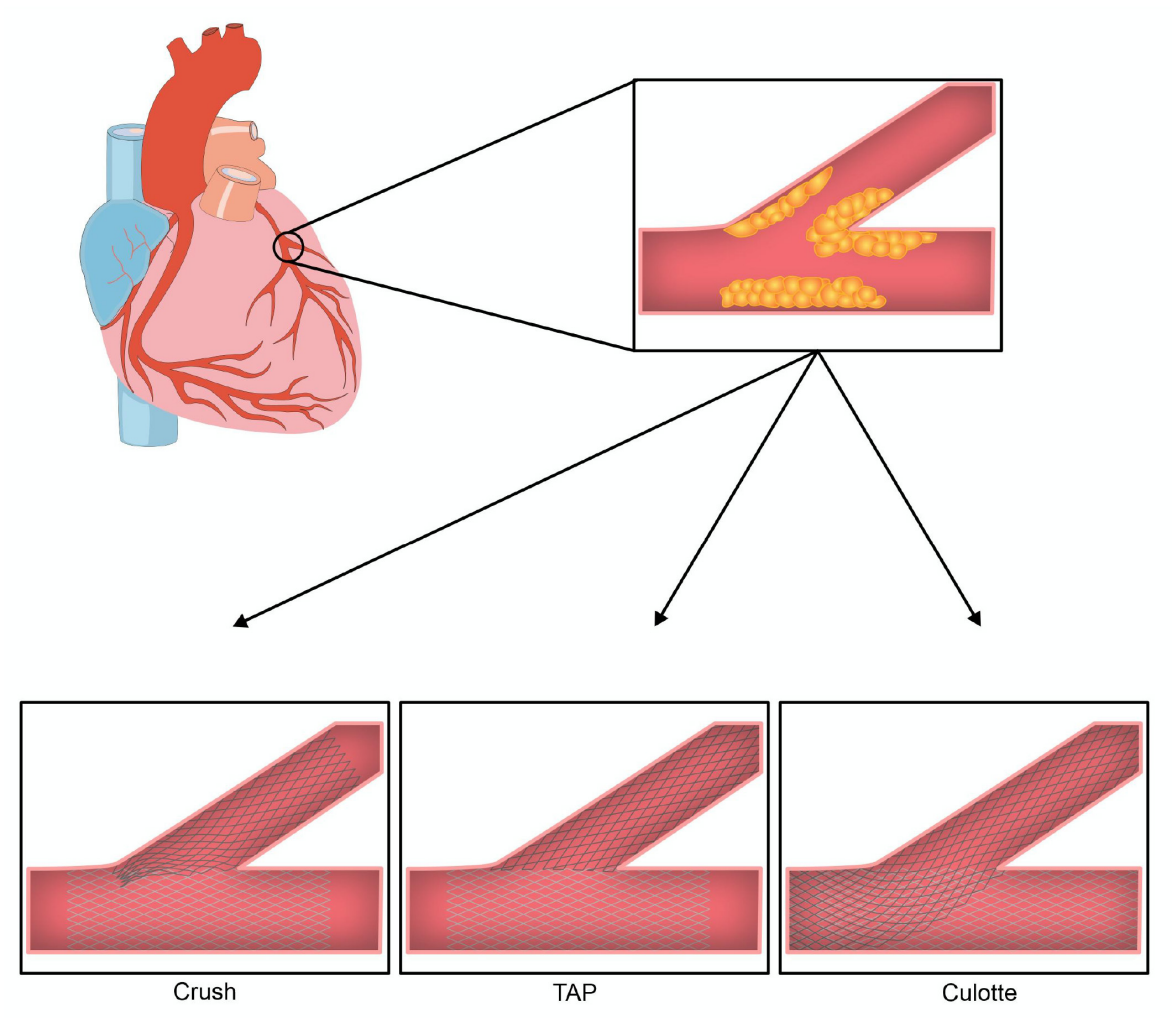
**The final two stent conformation in bifurcation lesions based on approach**. **Crush: **The first stent is placed in the side branch with protrusion into the main vessel. The protruding portion is ‘crushed’ against the wall by dilatation with a balloon with subsequent stent placement in the main vessel. The procedure is completed with a final kissing balloon. **TAP: **The first stent is placed in the main vessel followed by rewiring of the side branch and kissing balloon to open the ostium. Subsequently, the side branch stent is deployed with minimal protrusion and a final kissing balloon is performed. **Culotte: **The first stent is placed in the side branch with protrusion into the main vessel. The main vessel is rewired through the strut of the first stent. A second stent is then placed in the main vessel and finished with a final kissing balloon.

The provisional technique can be converted to a two-stent strategy with an additional stent in the side branch if there is a residual stenosis that can lead to significant ischemia, if there is compromised flow in the side branch or if there is side branch dissection. This can be achieved with T and small Protrusion (TAP) stenting, Culotte technique stenting or reverse Crush stenting [15]. In the TAP technique, the side branch stent protrudes completely into the carina to allow for full coverage of the carina. The Culotte technique provides complete coverage, as the side branch stent extends more fully into the main vessel. It is most appropriate for bifurcations with more shallow angles, although it may be limited by the number of times it requires rewiring [17]. In the reverse Crush technique, the second stent is deployed in the side branch, protruding 2–3 mm into the main vessel, followed by noncompliant inflation in the main vessel to flatten the side branch stent against the main vessel wall [18]. Routine kissing balloons should be used when two stents are inserted [3, 19]. Fig. 2 provides an illustration of the end formation of each of these techniques.

The 2011 ACC/AHA/SCAI guidelines and the EBC still recommend a provisional stenting technique for the majority of lesions [15, 20]. The 2021 ACC/AHA/SCAI guidelines did not comment on the optimal bifurcation stenting technique [21]. Notable landmark trials that have not shown any benefit of dedicated two-stent techniques include the Nordic Bifurcation Study, the British Bifurcation Coronary Study, the CACTUS (Coronary Bifurcations: Application of Crushing Technique using Sirolimus-eluting stents) study, and the Nordic-Baltic Bifurcation Study IV [22, 23, 24, 25]. A meta-analysis investigating differences between provisional stenting and up-front two-stent interventions found no benefit to the two-stent approach [26]. Additionally, a combined analysis of the Nordic Bifurcation Study and the British Bifurcation Coronary Study showed a lower 5-year survival as well as an increase in fluoroscopy time, contrast volume, and higher peri-procedural biomarkers in up-front two-stent interventions [27].

There may, however; be a role for an up-front two-stent approach in certain bifurcation lesions. The DEFINITION (Definitions and Impact of Complex Bifurcation Lesions on Clinical Outcomes After Percutaneous Coronary Intervention Using Drug-Eluting Stents) study proposed indications including a side branch with a diameter stenosis >70% and a lesion length of >10 mm or two minor criteria including moderate-severe calcification, multiple lesions, LAD-LCx bifurcation angle >70, main vessel diameter <2.5 mm, thrombus-containing lesions and main vessel length >25 mm. These criteria were validated in the initial cohort study. In a secondary analysis of lesions that met the complex criteria, the up-front two-stent approach had an overall lower incidence of major adverse cardiovascular events (MACE) and target lesion failure compared to provisional stenting [28].

These criteria were subsequently confirmed in the DEFINITION II RCT, which demonstrated a decrease in target lesion revascularization (TLR) and target vessel myocardial infarction (MI) in the two-stent cohort [29]. The study, however; did include mandatory angiography at 12 months which may have led to an increase in TLR at this time-point. Therefore, there were potentially more sub-clinical target lesion restenosis in the provisional cohort that were identified in the clinical trial that may otherwise not have been identified in clinical practice. Likewise, target vessel MI occurred in the provisional stent cohort mostly at the time of the index procedure and may be due to restrictive optimization of the side branch for provisional stenting dictated by the study protocol. Nevertheless, the DEFINITION studies provide a framework for a “lesion-specific” individual approach to a heterogenous group of bifurcation lesions that offers clinicians reasonable support for a dedicated two-stent technique.

In scenarios where the up-front two-stent technique is used, the crush techniques are preferable (Fig. 2) [30]. This recommendation is based on the DKCRUSH (Double Kissing Crush versus Provisional Stenting Technique for Treatment of Coronary Bifurcation Lesions) II trial, which showed superiority of the DK crush technique to provisional stenting in non-left main bifurcations [31]. While this study provides evidence that the DK Crush technique may be preferable, other derivations of the crush technique, including the Mini Crush technique, as well as the Culotte technique, Simultaneous Kissing Stents, V Stenting, T Stenting or TAP may also be considered in the appropriate clinical scenario (Fig. 2).

Currently, ESC guidelines recommend a two-stent approach if the scenario includes a large side branch with a long ostial side branch lesion or a distal left main (LM) bifurcation, or if difficulty is anticipated in accessing an important side branch after main branch stenting [32]. The European Bifurcation Club acknowledges a two-stent technique may benefit lesions involving a large and significantly diseased side branch [15].

### 3.2 Distal Left Main Bifurcation

Additional considerations need to be taken for LM bifurcation lesions as opposed to non-LM bifurcations. Specific considerations for LM bifurcations include the relatively large diameter of the vessel, the frequency of trifurcation lesions, and the atherosclerosis pattern which tends to be longitudinal and diffuse extending into both branches [33]. Perhaps the most relevant consideration is that the side branch is frequently the Left Circumflex artery (LCx), which is unique in its wide angulation and the relatively large amount of myocardium it supplies [33].

Due to these differences, several technical considerations need to be considered. First it is generally recommended to wire both branches before balloon dilatation for protection [33]. If LM coverage can be achieved with the same stent that is implanted in the Left Anterior Descending artery (LAD) or LCx, then the stent should be sized based on the diameter of the distal vessel and the POT should be used to achieve proper apposition within the LM [33]. The 13th and 14th consensus documents by the European Bifurcation Club also recommend the provisional technique for LM bifurcation disease that does not include both branches [33, 34]. If bail-out side branch stenting is needed, T-stent, TAP or Culotte stenting are preferred [33, 34].

If LM bifurcation disease involves both branches, the stent technique should depend on the coronary anatomy and the operator’s skill [33]. If an up-front two-stent strategy is pursued, evidence favors using the DK crush technique if technically feasible. This recommendation is based on the results of the DKCRUSH-III and DKCRUSH-V trials which showed the benefit of the DK crush technique for LM bifurcation stenting as compared to the Culotte technique and provisional strategy, respectively [35, 36]. Other recent trials have still shown the benefit of provisional stenting [37]. The proper approach to LM bifurcation stenting is not yet clearly defined and remains open for debate.

### 3.3 Additional Considerations in Bifurcation Lesions

It is unclear if there is any role for dedicated devices in bifurcation lesions in the current era. Most notably, in 2015, there was a study using a Tryton Side Branch Stent, a bare-metal stent (BMS), that had inferior outcomes compared to provisional stenting [37]. Another tool that may be useful in the setting of bifurcation lesions is drug-eluting balloons (DEB). These devices can be incorporated into the single stent approach while still providing anti-restenosis drug delivery into side branches. They have been shown to provide acceptable rates of late lumen loss in management of side branch lesions [38]. A meta-analysis comparing DEB to traditional balloon angioplasty in the management of side branch lesions showed decreased rates of side branch lumen loss in the DEB arm [39]. Additionally, DEB may be efficacious in treatment of side branch stenosis in distal left main bifurcation disease [40]. Additional information on lesion-specific interventions, tips and management of complications can be found in the BifurcAID application [41]. Future studies in this field will hopefully determine which specific lesions dictate an up-front two-stent approach and the best two-stent technique to achieve success.

## 4. Unprotected Left Main

Left main coronary artery disease (LMCAD) is associated with adverse outcomes, and is considered unprotected if the diseased left main artery does not have a patent bypass graft to the LAD or LCx. Due to the large myocardial territory it covers, medical therapy is generally not recommended [42]. Previous guidelines recommend revascularization for LMCAD >50% or positive functional testing [20, 43]. The most recent ESC guidelines also recommend using intravascular ultrasound (IVUS) to assess the severity of unprotected LMCAD and the ACC/AHA/SCAI guidelines recommend using IVUS in scenarios of intermediate stenosis of the LMCAD to better define lesion severity [21, 32]. Historically Coronary Artery Bypass Graft (CABG) surgery has been the gold standard for therapy and still represents the standard of care for many cases of LMCAD, however in certain scenarios, PCI is an acceptable alternative.

A sub-study comparing PCI to CABG in LMCAD was included in the SYNTAX (Synergy between PCI with Taxus and Cardiac Surgery) trial. This trial used the currently obsolete Paclitaxel DES and combined multi-vessel CAD with LMCAD. Overall, CABG outperformed PCI largely due to an increase in the need for repeat revascularization. However, when PCI was compared to CABG in the LMCAD population stratified by SYNTAX score, there was no difference in outcomes in the low and intermediate SYNTAX score cohorts; while CABG outperformed PCI in the high SYNTAX score cohort. When LMCAD was an isolated lesion, the initial and long term follow up trials did not show any overall difference in the primary endpoint between PCI and CABG [9, 44].

Subsequently, the PRECOMBAT (Premier of Randomized Comparison of Bypass Surgery versus Angioplasty Using Sirolimus-Eluting Stent in Patients with Left Main Coronary Artery Disease) trial and NOBLE (Nordic–Baltic–British Left Main Revascularization) trial evaluated PCI compared to CABG in isolated LMCAD. The PRECOMBAT trial used first generation DES and showed no difference between CABG and PCI, even when stratified by SYNTAX score [45]. The NOBLE trial used second generation DES and PCI and did not meet non-inferiority to CABG driven by MI and the need for repeat revascularization [46].

The most recent study to evaluate LMCAD was the EXCEL (Evaluation of XIENCE versus Coronary Artery Bypass Surgery for Effectiveness of Left Main Revascularization) trial. This trial is notable as it made strong recommendations for more modern techniques in both surgical and percutaneous intervention, such as transesophageal echocardiography, arterial revascularization and off-pump surgery as well as contemporary generation stents. Additionally, the trial was powered such that the need for repeat revascularization was not included in the primary outcome. This study did not find any difference in outcomes of death, stroke or MI at up to 5 years. When stratifying outcomes by time after intervention, PCI was associated with improved outcomes within the first 30 days and equivalent outcomes between 1 month and 1 year as compared to CABG. CABG demonstrated a limited benefit compared to PCI from 1 to 5 years. Subgroup analysis did not find any significant differences by SYNTAX score [47, 48].

A recent meta-analysis comparing PCI to CABG for LMCAD did not find any difference in 5 year or 10 year all-cause death, although there was an increase in spontaneous MI and repeat revascularization in the PCI group. A majority of patients in the analysis had low to intermediate SYNTAX scores [49]. The 2018 ESC guidelines currently state PCI is an appropriate alternative to CABG in LMCAD with Syntax scores </ = 22 as well as Syntax scores between 22–32 and contraindicated in Syntax scores >32 [32]. Table 1 highlights both the initial and long-term outcomes from these trials [9, 44, 45, 46, 47, 48, 50, 51].

**Table 1.** **Comparison of randomized controlled trials for PCI vs CABG in treatment of left main coronary artery disease**.

	SYNTAX (2009)	PRECOMBAT (2011)	NOBLE (2016)	EXCEL (2016)
	n = 1800	n = 600	n = 1201	n = 1905
	Stent = Paclitaxel eluting stent	Stent = Sirolimus eluting stent	Stent = Biolimus eluting stent	Stent = Everolimus eluting stent
	(1st generation)	(1st generation)	(2nd generation)	(2nd generation)
Study Population	Untreated left main coronary artery disease AND triple vessel disease	Untreated left main coronary artery disease	Untreated left main coronary artery disease	Untreated left main coronary artery disease
	No upper limit of SYNTAX score	No upper limit of SYNTAX score	No upper limit of SYNTAX score	SYNTAX score 32 or lower
Primary Outcome	Composite of all-cause mortality, stroke, MI or unplanned revascularisation at 1-year follow-up	Composite of all-cause mortality, stroke, MI or unplanned revascularisation at 1-year follow-up	Composite of all-cause mortality, stroke, MI or unplanned revascularisation at 1-year follow-up	Composite of all-cause mortality, stroke or MI
Initial Outcomes	- Increase in primary outcome in PCI compared to CABG (17.8% vs 12.4% for CABG; *p* = 0.002)	- No difference in primary outcome in PCI compared to CABG (8.7% PCI vs 6.7% CABG *p* = 0.01 for non-inferiority)	- Increase in primary outcome in PCI compared to CABG (28% PCI vs 18% CABG *p* = 0.0044 for superiority)	- No difference in primary outcome in PCI compared to CABG (15.4% PCI vs 14.7% CABG *p* = 0.02 for non-inferiority)
	- Largest contributor to primary outcome was repeat revascularization (13.5% vs 5.9%, *p *< 0.001)	- Increase in target vessel revascularization in PCI compared to CABG (9% PCI vs 4.2% CABG at 24 months *p* = 0.02)	- Increase in both target vessel revascularization (15% PCI vs 10% CABG *p* = 0.0304) and MI (6% MI vs 2% CABG *p* = 0.0040) in PCI compared to CABG	- Decrease in death, stroke and MI at 30 days for PCI compared to CABG (4.9% vs 7.9% *p* = 0.008 for superiority)
	- Stroke was significantly more likely to occur with CABG (2.2% vs 0.6% with PCI; *p* = 0.003).	- No difference between PCI and CABG in low, intermediate and high SYNTAX score	- Patients in low SYNTAX score benefitted from CABG compared to PCI and there was no difference in intermediate and high SYNTAX score	
	- Patients in low and intermediate SYNTAX scores had similar rates of MACE between PCI and CABG			
Long Term Outcomes	10 year follow up (2019)	10 year follow up (2020)	5 year follow up (2020)	5 year follow up (2019)
	- No difference in all cause mortality in left main coronary artery disease between PCI and CABG (26.6% PCI vs 28.2% CABG; HR 0.92 [95% CI 0.69–1.22])	- No difference in primary outcome but trend toward benefit in CABG (29.8% PCI vs 24.7% CABG HR = 1.25 [95% CI, 0.93–1.69])	- Increase in primary outcome in PCI compared to CABG (28% PCI vs 19% CABG *p* = 0.0002 for superiority)	- No difference in primary outcome in PCI compared to CABG (22% PCI vs 19.2% CABG *p* = 0.13)
		- Largest contributor to primary outcome was repeat revascularization (16.1% PCI vs 8.0% CABG; HR 1.98 [95% CI, 1.21–3.21)	- Increase in both target vessel revascularization (17% PCI vs 10% CABG *p* = 0.0009) and MI (8% MI vs 3% CABG *p* = 0.0002) in PCI compared to CABG	- Increase in all cause mortality for PCI compared to CABG (13% PCI vs 9.9% CABG Absolute Difference = 3.1% [95% CI, 0.2%–6.1%]) but no difference in cardiovascular death (5.0% PCI and 4.5% CABG Absolute Difference = 0.5% [95% CI, −1.4%–2.5%)
		- No difference in all cause mortality (14.5% PCI vs 13.8% CABG; HR 1.13 [95% CI, 0.75–1.70])	- No difference in all cause mortality (9% PCI vs 9% CABG; HR 1.08 [95% CI, 0.74–1.59])	

### Unprotected Left Main Technical Considerations

If PCI is elected for LMCAD revascularization, technical considerations include the use of intravascular imaging and functional assessments, the use of hemodynamic support and management of bifurcation lesions. IVUS can help determine anatomy, plaque configuration and distribution of bifurcations. Typically, an IVUS minimal luminal area <6 ${\mathrm{mm}}^{2}$ has been used as a cutoff for clinically significant left main disease, although a smaller cutoff may be needed for patients of Asian descent [21, 32, 52]. By comparison, optical coherence tomography (OCT) is of more limited utility for LMCAD, particularly ostial LMCAD, because this technology requires blood clearance through the use of contrast injection [21]. Pooled analysis shows IVUS-guided left main interventions have demonstrated a mortality benefit compared to ‘unguided’ PCI [53]. Fractional flow reserve (FFR) can also be helpful in determining which unprotected distal left main lesions warrant intervention. A cutoff of >0.80 has been shown to have similar outcomes in medically treated patients compared with revascularization [54].

Identifying anatomy is of particular concern in LMCAD given differences in PCI outcomes between ostial or body lesions compared to distal bifurcation lesions. Ostial and body lesions account for approximately one third of LMCAD [55]. These lesions have more favorable outcomes and a lower incidence of restenosis [56]. While single stent insertion into ostial lesions can be straightforward, care must be taken not to overextend stents by more than 2 millimeters into the aorta to allow for subsequent re-engagement into the LM artery [57]. The specialized double ostial flash balloon can aid interventionalists in providing complete coverage of the aorto-ostial interface while also keeping the lumen patent for re-engagement. Bifurcation lesions, by comparison, represent the majority of LMCAD, are more technically challenging, and are associated with worse outcomes [56, 58]. Specific considerations for left main bifurcation lesions have been previously described.

Another consideration is whether the use of hemodynamic support during LM PCI is warranted, especially in scenarios of acute coronary syndrome (ACS) where patients with LM culprit lesions are more likely to present in cardiogenic shock [59]. Briguori *et al*. [60] evaluated prophylactic use of an intra-aortic balloon pump (IABP) in elective unprotected LM disease undergoing PCI and showed that the IABP group had lower intraprocedural events. SCAI and the ACC/AHA recommend considering MCS in those patients with reduced EF, decompensated hemodynamics, or expected prolonged ischemic times due to the anatomy of the lesions and the need for atherectomy [61].

There is unanimous support by both cardiothoracic surgeons as well as cardiologists to deploy a Heart Team approach to determine the best strategy for coronary revascularization in an individual patient. This multidisciplinary team should consider the patient’s comorbidities, SYNTAX score, Society of Thoracic Surgery (STS) risk score and patient preferences to determine the best intervention in each clinical setting [43].

## 5. Calcified Lesions

Coronary artery calcification carries significant challenges due to unique technical considerations of the procedure as well as a challenging patient population. Calcifications limit the effectiveness of balloon angioplasty as well as stent delivery and expansion. Additionally, patients with calcified lesions are more likely to be older and have more comorbidities including renal dysfunction, anemia and previous CABG [62, 63, 64, 65]. Calcifications have been shown to be an independent risk factor for stent failure and subsequent ischemic events and are independently associated with MACE including death following PCI [66, 67, 68, 69]. Newer generation DES are engineered to be less thrombogenic and have been shown to be superior to previous generations of DES and BMS in the management of calcified lesions [62, 70].

### Atherectomy, Lithotripsy and Specialized Balloon Angioplasty

Calcium modification techniques such as specialized balloons, atherectomy, lithotripsy, and laser technology can improve stent deployment and outcomes (Table 2). Traditional balloon angioplasty can lead to coronary dissection or perforation if applied to calcified lesions because of the non-homogenous nature of calcific plaques. Ultra-high pressure noncompliant balloons have been developed that allow for more symmetrical expansion and small studies have shown that they provide a high degree of procedural success and low rates of perforation in calcified lesions [71, 72].

**Table 2.** **Pros and Cons of tools used in the management of calcified lesions**.

	Orbital and Rotation atherectomy	Lithotripsy	Ultra-high pressure non-compliant balloons	Cutting and scoring balloons
	Orbital and Rotation atherectomy	Lithotripsy	Ultra-High Pressure Non-Compliant Balloons	Cutting and Scoring Balloons
Pros	- Most well-studied and validated	- Potentially less damage to vessel walls compared to atherectomy	- More uniform coverage compared to standard balloons	- More controlled cutting
				- Increased lumen gain compared to standard balloon
Cons	- Complexity and cost	- Unclear long term outcomes	- Inability to recross when inflated due to the high profile and stiffness of twin-layer technology	- Increase risk of perforation
	- Advanced operative experience			- Can be difficult to deliver because of blade rigidity
				- Improved side effect profile in scoring balloon but unclear efficacy

Cutting and scoring balloons have also been developed to break calcium as they expand. Cutting balloons are an older technology, and evidence to support its use is mixed as some studies have shown limited efficacy with higher rates of perforation [73, 74]. Scoring balloons are thought to be a safer option and enhance stent expansion compared to traditional balloon angioplasty with an acceptable side effect profile [75]. Both the ESC and ACC/AHA/SCAI offer limited guidance on the use of these technologies [21, 32].

Atherectomy can remove calcified plaque and aid in stent delivery and expansion. Presently there are two types of atherectomy devices available: rotational (RA) and orbital (OA) atherectomy. Rotational devices have been generally more studied. The ROTAXUS (Rotational Atherectomy Prior to TAXUS Stent Treatment for Complex Native Coronary Artery Disease) trial illustrated improved procedural success associated with RA compared to stenting without atherectomy but did not show any difference in 9 month outcomes [76]. OA devices were evaluated in the ORBIT II (Evaluate the Safety and Efficacy of OAS in Treating Severely Calcified Coronary Lesions) trial, which achieved its pre-specified safety and efficacy endpoints with sufficient freedom from 30 day MACE and low rates of residual stenosis [77, 78].

A systematic review and meta-analysis comparing OA and RA yielded similar results [79]. At this time the choice between which device to use should be based on user preference and institutional availability. Current recommendations by the ACC/AHA/SCAI suggest RA in scenarios of fibrotic or heavily calcified plaques to improve procedural success. However, new indications for use are currently being investigated. Lee *et al*. [80] advocates atherectomy use for severe coronary artery calcification (CAC) by angiography and further evaluation with IVUS and OCT for moderate CAC. Atherectomy should be used if CAC is greater than 270° and considered if CAC is 180°–270°. Additionally, calcium scoring systems using IVUS and OCT have been developed that can help predict stent under-expansion and may guide operators on when to perform atherectomy [81, 82]. Intravascular imaging also provides information on calcium length and thickness, which are predictive of procedural success and can guide operators on which tools may be most effective [83].

Laser atherectomy (ELCA) is another alternative for calcifications however early studies showed that it was inferior compared to balloon angioplasty alone [84, 85]. It may be helpful to treat lesions that lead to stent under-expansion that cannot be dilated with high-pressure balloons, however ELCA tends to be ineffective for severe calcification [21, 80]. The use of ELCA along with iodinated contrast injection for treatment of focal calcific lesions has also been described.

Intravascular lithotripsy is another contemporary option which uses pressure waves that fracture the intimal and medial wall calcium. The Disrupt-CAD III (Disrupt Coronary Artery Disease) trial proved that it is a safe and effective modality for intervening on calcium plaques. It should be noted however, that long-term safety and efficacy have yet to be determined and long-term outcomes from the initial trauma are unknown [86, 87].

## Abbreviations

ACC, American College of Cardiology; AHA, American Heart Association; ACC, American College of Cardiology; ACS, Acute Coronary Syndrome; AHA, American Heart Association; BMS, Bare Metal Stent; CABG, Coronary Artery Bypass Graft; CAC, Coronary Artery Calcification; CACTUS, Coronary Bifurcations, Application of Crushing Technique using Sirolimus-eluting stents; CAD, Coronary Artery Disease; CHIP-PCI, Complex high-risk indicated percutaneous coronary intervention; DEB, Drug-Eluting Balloon; DEFINITION, Definitions and Impact of Complex Bifurcation Lesions on Clinical Outcomes After Percutaneous Coronary Intervention Using Drug-Eluting Stents; DES, Drug Eluting Stent; Disrupt-CAD III, Disrupt Coronary Artery Disease; DKCRUSH, Double Kissing Crush versus Provisional Stenting Technique for Treatment of Coronary Bifurcation Lesions; ESC, European Society of Cardiology; FFR, Fractional Flow Reserve; IABP, Intra-aortic Balloon Pump; IVUS, Intravascular Ultrasound; LAD, Left Anterior Descending artery; LCx, Left Circumflex artery; LM, Left Main artery; LMCAD, Left Main Coronary Artery Disease; MACE, Major Adverse Cardiovascular Events; MI, Myocardial Infarction; NOBLE, Nordic–Baltic–British Left Main Revascularization; OA, Orbital Atherectomy; OCT, Optical Coherence Tomography; ORBIT II, Evaluate the Safety and Efficacy of OAS in Treating Severely Calcified Coronary Lesions; PCI, Percutaneous Intervention; POT, Proximal Optimization Technique; PRECOMBAT, Premier of Randomized Comparison of Bypass Surgery versus Angioplasty Using Sirolimus-Eluting Stent in Patients with Left Main Coronary Artery Disease; RA, Rotational Atherectomy; RCT, Randomized Controlled Trial; ROTAXUS, Rotational Atherectomy Prior to TAXUS Stent Treatment for Complex Native Coronary Artery Disease; SCAI, Society for Cardiovascular Angiography and Intervention; STS, Society of Thoracic Surgery; SYNTAX, Synergy between PCI with Taxus and Cardiac Surgery; TAP, T and small protrusion.
